# Strategy of skull base reconstruction after endoscopic transnasal pituitary adenoma resection

**DOI:** 10.3389/fsurg.2023.1130660

**Published:** 2023-03-14

**Authors:** Chao Zhang, Zhijun Yang, Pinan Liu

**Affiliations:** ^1^ Department of Neurosurgery, Beijing Tiantan Hospital, Capital Medical University, Beijing, China; ^2^ Beijing Neurosurgical Institute, Capital Medical University, Beijing, China

**Keywords:** CSF leakage, neuroendoscopy, basicranial tumor, skull base reconstruction, fascia sutured

## Abstract

**Objective:**

Endoscopic endonasal surgery (EES) is commonly performed for resection of lesions of the anterior/middle cranial fossa region. Cerebrospinal fluid (CSF) leakage is a major complication. Skull base reconstruction after EES is challenging. We describe our reconstruction strategy and technique and analyze its outcomes.

**Methods:**

We retrospectively analyzed 703 patients with pituitary adenoma who underwent EES in our center from January 2020 to August 2022. Clinical, imaging, operative, and pathologic data were recorded from the medical records and analyzed. Skull base reconstruction was performed to achieve the following three goals: seal the original leak, eliminate dead space, provide blood supply, and early ambulation. Reconstruction was tailored to individual patients based on grade of CSF leakage encountered during surgery.

**Results:**

The number of patients with a grade 0, 1, 2, and 3 intraoperative CSF leak was 487, 101, 86, and 29, respectively. Overall incidence of postoperative CSF leakage was 0.14% (1/703). Fascia sutured and vascularized nasoseptal flap were selected for all grade 3 CSF leaks. One patient who experienced postoperative CSF leakage developed intracranial infection and were treated with lumbar CSF drainage that failed; eventually re-exploration surgery for repair was required. Other patients did not have complications such as CSF leak and infection. 29 patients with grade 3 CSF leakage did not complain of severe nasal complications after operation. No perioperative complications related to the strategy (overpacking, infections, or hematomas) occurred. Incidence of postoperative CSF leak according to intraoperative leak grade was as follows: grade 0, zero; grade 1, zero; grade 2, 1.16% (1/86); and grade 3, zero.

**Conclusion:**

The principles of sealing the original leak, eliminating dead space, providing blood supply, and early ambulation are key in skull base reconstruction after EES. Individualization of these principles can significantly reduce the incidence of postoperative CSF leakage and intracranial infection and reduce the use of lumbar CSF drainage. Skull base suture technique is safe and effective in patients with high-flow cerebrospinal fluid leaks.

## Introduction

Endoscopic endonasal surgery (EES) is widely used for resection of basicranial tumors (1–3). Cerebrospinal fluid (CSF) leakage is a common complication, as the approach entails removal of skull base bone and entering the subarachnoid space. Reported incidence rates of CSF leakage after EES range from 1.6% to 40% (4–6). In our previous study, intraoperative CSF leakage was the only predictor of postoperative leakage (7). Rigorous intraoperative skull base reconstruction is essential for prevention.

In 2006, Hadad et al. first described using a vascularized nasoseptal flap for skull base reconstruction, which substantially reduced the incidence of postoperative CSF leakage (8). A system of classification for CSF leaks and their corresponding repair methods was proposed in 2007 (9). Improvements in technique since then, including buttressing and increased use of vascularized pedicle flaps, have resulted in lower rates of postoperative CSF leakage (10). In patients deemed at high risk of CSF leak, use of a vascularized nasoseptal flap and perioperative lumbar CSF drainage reduces the CSF leak rate (11).

Despite these improvements, postoperative CSF leakage may still occur. To further decrease the incidence of CSF leakage in our center, we implemented a new skull base reconstruction strategy in 2020 based on three principles: sealing the original leak, eliminating dead space, and providing blood supply. In this report, we describe our reconstruction strategy and technique and analyze its outcomes.

## Methods

The inclusion criteria were endoscopic surgery and pathological diagnosis of pituitary adenoma. We retrospectively analyzed 703 patients in our center from January 2020 to August 2022. Clinical, imaging, operative, and pathologic data were recorded from the medical records and analyzed. Postoperative CSF leakage, intracranial infection, and nasal quality of life were the indicators we focused on. All patients were followed up by telephone at 3 and 6 months after surgery to ask whether the above complications occurred. The study was approved by the ethics committee of Beijing Tiantan Hospital, Capital Medical University. All patients provided informed consent.

### Principles of skull base reconstruction

#### Sealing the original leak

The goal of sealing the original leak is to achieve the same cranial physiological state as before surgery where possible and prevent CSF leakage. We believe that this principle is key when reconstructing the skull base.

#### Eliminating dead space

Filling any dead space with repair material can protect the dura of the diaphragma sellae and prevent empty sella syndrome.

#### Providing blood supply

The vascularized nasoseptal flap, contralateral rescue flap, and pedicled middle turbinate mucosal flap provide blood supply to the reconstructed area. Considering the impairment of olfactory function, we prepared mucosal flaps only for patients deemed likely to experience high-flow (grade 3) CSF leakage based on preoperative assessment. These included patients with a pituitary adenoma classified as Hardy–Wilson grade B, C, or D (12).

#### Early ambulation, rapid recovery

After surgery, patients were encouraged to stand and walk as much as possible to shorten the recovery time.

### Method of skull base reconstruction

#### Grade 0 (no intraoperative CSF leak)

With grade 0 CSF leakage, the diaphragma sellae is intact and CSF leakage is not observed. The general principle is to restore the normal structure of the saddle area using fibrin glue to strengthen the diaphragma sellae, then packing gelatin sponge within the sella.

#### Grade 1 (small weeping leak without obvious defect)

**Figure 1:**
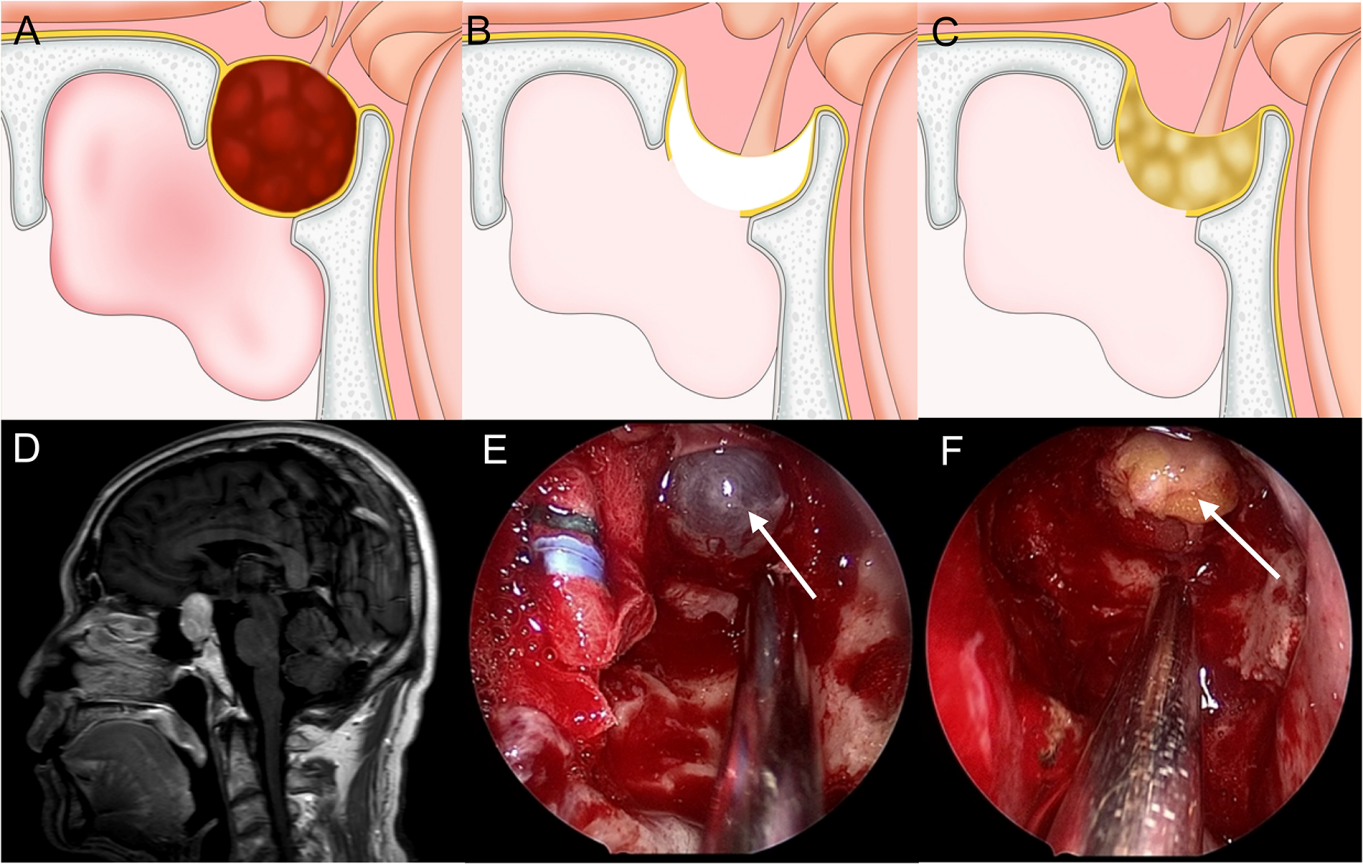
Method for repairing a grade 1 CSF leak. (**A,D**) Sagittal view: the tumor is mainly within the sella and the diaphragma sellae is slightly upward. (**B,E**) The diaphragma sellae is descending and thin with CSF exudation. (**C,F**) Fat is packed into the saddle to seal the leak and eliminate dead space.

Although no diaphragma sellae damage is seen in grade 1 CSF leaks, a small amount of CSF leakage is seen. The principles of repair are reinforcement of the original leak and elimination of dead space by packing fat within the sella, which can achieve both. The artificial dura is then attached to the outer layer of fat, fixed with a thin layer of fibrin glue to strengthen the sellar dura, and the sphenoid sinuses are then filled with absorbent cotton (Figure 1).

#### Grade 2 (moderate leak)

**Figure 2:**
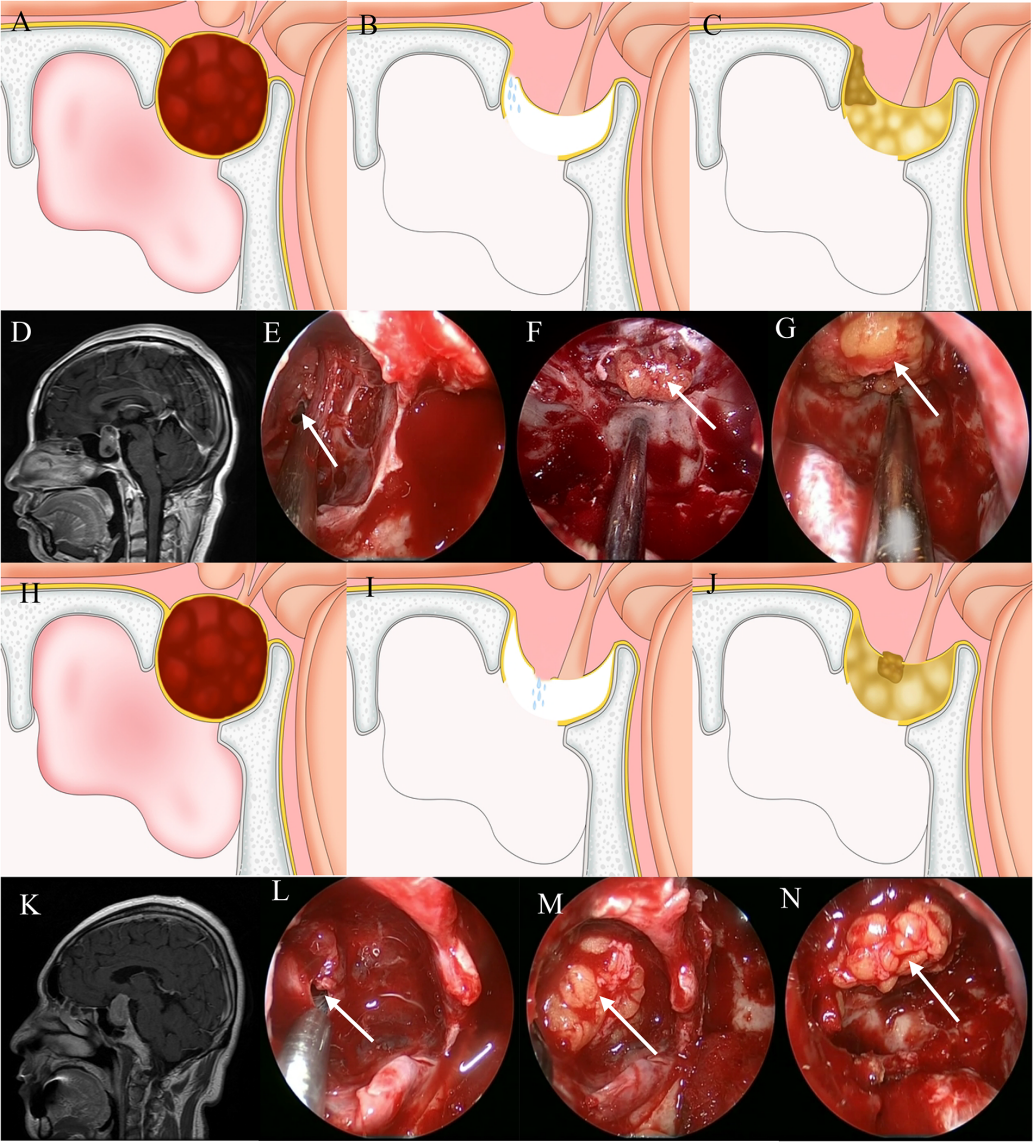
Method for repairing a grade 2 CSF leak. (**A,D**) Sagittal view: the tumor exhibits suprasellar invasion and the diaphragma sellae is elevated. (**B,E**) A CSF leak is seen at the junction between the diaphragm sellae and intrasellar dura. (**C,F**) A suitably sized fat block “bathtub plug” type sealing the leak. (**G**) Fat is packed into the saddle to eliminate dead space. (**H,K**) Sagittal view: the tumor exhibits suprasellar invasion and the diaphragma sellae is elevated. (**I,L**) A CSF leak is seen along the sagging portion of the diaphragma sellae. (**J,M**) A suitably sized fat block “bathtub plug” type sealing the leak. (**N**) Fat is packed into the saddle to eliminate dead space.

Grade 2 CSF leakage is defined as a significant leak at the diaphragma sellae. The principles of repair are sealing the original leak and eliminating dead space by filling the damaged area of the diaphragma sellae with an appropriately sized autologous fat graft and keeping the diaphragma sellae intact with a “bathtub plug.” Then, the sella is filled with autologous fat to eliminate dead space. Then fibrin glue was used to fix an artificial dural on the outside of the fat. A vascularized nasoseptal flap is not used. After repair, the nasal passage is filled with iodoform gauze that is removed 1 week after surgery (Figure 2).

#### Grade 3 (large defect)

**Figure 3:**
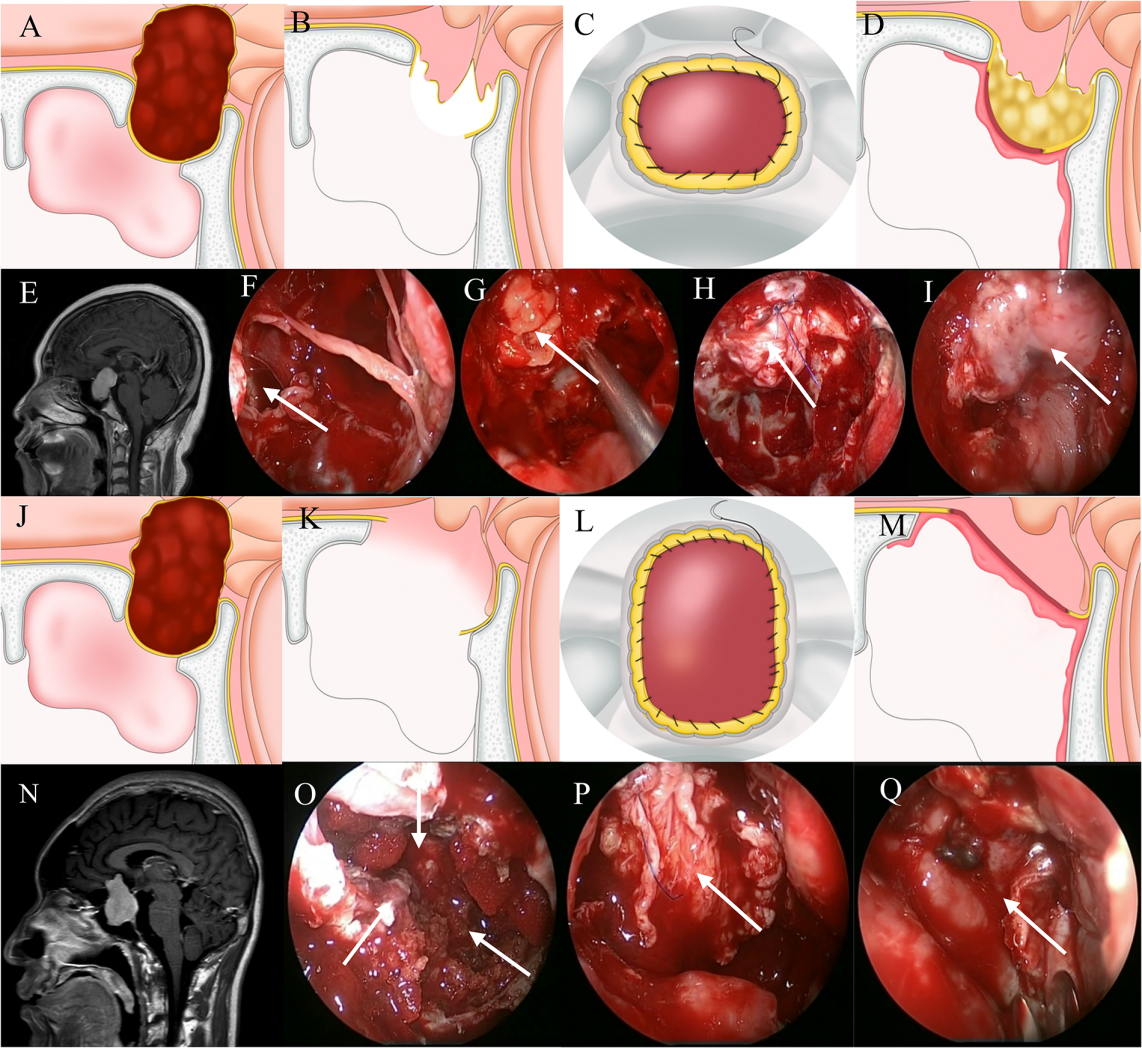
Method for repairing a grade 3 CSF leak. (**A,E**) Sagittal view: the tumor is located predominantly above the sella. (**B,F**) A large leak through the diaphragmatic sellae is seen. (**G**) Fat is packed into the saddle to eliminate dead space. (**C,H**) Autogenous fascia and sellar dura are sutured continuously. (**D,I)** A vascularized nasoseptal flap provides blood supply. (**J,N**) Sagittal view: the tumor is located predominantly above the sella. (**K,O**) The diaphragma sellae is badly damaged. (**L,P**) Autogenous fascia and skull base dura are sutured continuously. (**M,Q**) A vascularized nasoseptal flap provides blood supply.

With a grade 3 CSF leak, a large defect in the diaphragma sellae and/or anterior skull base dura has been created as part of the surgical approach and the floor of the third ventricle is open. Therefore, the degree of CSF leakage is large. We classify grade 3 CSF leaks into two subtypes. In the first, the planum sphenoidale dura has been opened and the sellar region structure has been completely destroyed. In the second, “fishnet” CSF leakage from the diaphragma sellae occurs while the structure of the sellar region has remained intact. For repair of the first type, autologous fascia is continuously sutured with the dura of the anterior skull base. For the second, the first step is filling the sella with autologous fat to eliminate dead space followed by continuous suturing of autologous fascia with saddle bottom dura mater to seal the leak. Then, a vascularized nasoseptal flap is placed. After repair, the nasal passage is filled with iodoform gauze that is removed after 7–10 days (Figure 3).

### Suture technique

continuous suturing of the dura with the fascia lata grafts, was performed using 5–0 resorbable sutures (polydioxanone monofilament sutures: PDS®; Ethicon, Somerville, NJ, United States). The key step of the skull base suture technique is how to end the operation as soon as possible while ensuring the watertightness. We will introduce our surgical techniques. First, we will cut the appropriate size of autogenous fascia and place it at the “3 o'clock position” of the sellar dura mater defect as the first suture stitch. The direction of the fascia is from outside to inside. The injection direction of the dura mater is from the inside to the outside, and the knot of the first needle must be absolutely firm. Suture continuously counterclockwise, every 2–3 stitches, tighten the suture to prevent the knot from loosening. After sewing a circle, the last stitch is tied with the first stitch. The needle distance is about 2–3 mm, and care should be taken not to damage the bilateral internal carotid arteries when suturing the dura mater on both sides. We believe that the advantage of continuous suture is that it can shorten the operation time and reduce the difficulty of operation caused by multiple knots. But its disadvantage is that it is necessary to keep the suture in a tight state to prevent the knot from slipping, which undoubtedly increases the difficulty of cooperation between the surgeon and the assistant. Another key operation of skull base suture is knotting, the knot was made outside the nasal cavity and then slid into the operative region.

### Postoperative care and evaluation

We did not routinely use lumbar CSF drainage. Patients were kept in bed on the day of surgery. On the day after, posture and diet were not restricted. Patients were instructed to avoid actions that increase intracranial pressure. The nasal cavity was cleaned approximately 1 week after surgery to prevent nasal mucosal adhesions that can result in olfactory dysfunction.

Intravenous antibiotics (ceftriaxone 2 g daily or cefuroxime 1.5 g twice daily) were administered for 48 h after surgery. Patients with signs of infection were treated according to results of biochemical CSF testing. For patients with a postoperative CSF leak, surgical exploration was performed if an intraoperative repair had not been performed. In those whom intraoperative repair had been performed, a lumbar drain was placed; if leakage persisted more than 3 days, surgical repair was performed.

## Results

No after operative complications related to the strategy (overpacking, infections, or hematomas) occurred. Patient characteristics are shown in Table 1. CSF leaks were repaired according to grade as described above.

**Table 1:** Characteristics of the study population.

Characteristics	Value
Mean age (range), years	46.6 (33–60)
Males/females	329/374
**Pathology**
Nonfunctioning pituitary adenoma	621
Prolactinoma	17
Growth hormone–producing pituitary adenoma	65
Total	703

Overall, one patient experienced postoperative CSF leakage and infection (0.14%). In each, lumbar CSF drainage was ineffective and additional exploratory repair was required. The occurrence of CSF leakage according to pathological tumor type is shown in Table 2. Incidence of postoperative CSF leak according to intraoperative leak grade was as follows: grade 0, zero; grade 1, zero; grade 2, 1.16% (1/86); and grade 3, zero.

**Table 2:** The occurrence of CSF leakage during operation.

Pathology	Value
**Pituitary adenoma**
Grade 0	487
Grade 1	101
Grade 2	86
Grade 3	29

Table 3 summarizes the patient who experienced postoperative CSF leakage. The first was a 68-year-old woman with a pituitary adenoma. During surgery, a grade 2 CSF leak was encountered and repaired. Postoperative CSF leakage began 49 days after surgery. During reoperation, the sellar floor dura mater was encased and eroded by inflammatory exudate and the diaphragmatic foramen was enlarged. The leak was repaired using a “bathtub plug” and dead space was filled with autologous fat. Then, attach the local tissue with vascular pedicled nasal septum mucosal flap. Finally, fibrin glue was sprayed on the surface. Unfortunately, 43 days later, another CSF leak developed. Re-exploration was performed, which revealed CSF leaking from the diaphragmatic foramen. After repairing the leak with autologous fat and eliminating dead space, autologous fascia was used to suture the sellar floor dura mater continuously and attach local tissue with a vascularized nasal septum mucosal flap. Fibrin glue was applied again.

**Table 3:** The cases of CSF leakage after surgery.

Pathology	1st Operation		After 1st operation		2nd Operation	3rd Operation
	Leakage grade	Nasoseptal flap	Intracranial infection	Lumbar drainage		
Pituitary adenoma	2	No	Yes	Yes	Fat; autologous fascia; nasoseptal flap; fibrin glue	Fat; continuous suturing of the autologous fascia; nasoseptal flap; fibrin glue

In our opinion, for patients with po-CSF leakage, we reviewed the procedure of the second and third operations. At the second operation, we found an inflammatory discharge covering the dural at the base of the saddle and evidence of local infection. Therefore, we believe that the main causes of CSF leakage after the patient's first operation are poor local healing and local infection. At the same time, we also believe that the patient's emotional instability will lead to intracranial elevation, which is also the cause of po-CSF leakage. For patients with grade 2 CSF leakage during the operation, the size of the fat mass is the key to determine the success of the repair of CSF leakage. If the fat mass is too small, it is easy to cause po-CSF leakage. If the fat block is too large, it is prone to the occupying effect. After the appropriate size of the fat blocked the leak, the fat block could be seen pulsating with the pulse of the CSF. During the third operation, we found no obvious signs of infection locally, but there was a gap between the fat mass and the leakage. In order to prevent the recurrence of CSF leakage completely, we chose continuous suture as the repair method. Now, our retrospective analysis suggests that higher levels of repair should be selected when patients first experience CSF leakage after surgery.

## Discussion

EES plays an important role in the treatment of basicranial tumors. However, it requires skull base reconstruction, which can be challenging. The most serious complication of EES is CSF leakage. Risk factors include intraoperative CSF leakage and skull base defect location and size. The proposed reconstruction strategies described above, which are based on the grade of intraoperative CSF leak encountered, are expected to further decrease the incidence of CSF leakage and use of lumbar drainage.

### The development of skull base reconstruction

In recent years, the incidence of CSF leak after EES has decreased significantly in conjunction with advances in skull base reconstruction technology and techniques (Table 4). However, CSF leaks are still a major cause of prolonged hospital length of stay. Moreover, they may result in infection of the central nervous system and even death. Esposito et al. advocated tailoring the repair according to type of intraoperative CSF leak and dural defect (9). Solari et al. proposed that the “3F” reconstruction strategy of skull base, namely Fat, Flap and Flash, achieved good reconstruction results (13), and we fully agree with the above two reconstruction strategies. Our approach, which we have been using since 2020, differs somewhat and is based on sealing the intraoperative leak, eliminating dead space, providing blood supply, and early ambulation. Furthermore, our approach is individualized according to grade of intraoperative CSF leak.

**Table 4:** Literature review of repair of anterior skull base reconstruction.

Authors and year	Repair method	Other technique	Postoperative CSF leakage				
			Total	Grade 0	Grade 1	Grade 2	Grade 3
Esposito et al., 2007	Fat, collagen sponge	LD (Grade 3)	2.5% (17/668)	0.7% (2/290)	3% (7/217)	1% (1/103)	12% (7/58)
Kassam et al., 2008	Nasoseptal flap	Foley catheter					10.7% (8/75)
Zanation et al., 2009	Nasoseptal flap						5.7% (4/70)
Rosen et al., 2010	Button (fascia lata)	LD, NS flap					10% (4/40)
Garcia-Navarro et al., 2013	Gasket seal closure	Fat graft, NS flap, LD, tissue sealants					4.3% (2/46)
Hara et al., 2015	Dural suture, Fat, sphenoidal sinus mucosal flap	Fascia suturing (Grade 3)	1.0% (2/194)	0% (0/69)	0% (0/51)	0% (0/30)	4.5% (2/44)
Amano et al., 2016	Dural suturing, sphenoid sinus mucosal flap	Fat graft	1.2% (6/500)	NA	NA	NA	NA
Ishikawa et al., 2018	Continuous dural suturing, fat graft	Lactate plate, nasoseptal flap (Grade 3)	2.2% (4/176)	NA	1% (1/100)	2.9% (1/34)	4.7% (2/42)
Present study	Fat, bathtub plugs, continuous dural suturing	Nasoseptal flap (Grade 3)	0.14% (1/703)	0% (0/487)	0% (0/101)	1.16% (1/86)	0% (0/29)

NA, not available.

### Sealing the original leak

Isolating the extracranial space from the intracranial space is fundamental to prevent CSF leakage and intracranial infection and is the theoretical basis of repair. Early in our experience, CSF leak treatment was usually coverage or with repair material. However, this method does not achieve physiological closure of the leak. We now consider sealing the original leak to be the key factor in repair (**Grade 1**: packing fat within the sella; **Grade 2**: fat “bathtub” plug; **Grade 3**: autologous fascia suturing). The fat plays a very important role in the “3F” reconstruction strategy proposed by Solari et al., which is consistent with our chosen repair strategy in grade 1–2 CSF leakage, that fat can seal the leak like a “cork”. More importantly, the fat can be plastic in the surgical cavity, thus filling some irregular cavities. Different from the “3F” strategy, we chose a continuous suture method for the repair of grade 3 CSF leakage. In our center, we previously conducted a retrospective analysis on patients with high-flow CSF leakage during surgery to discuss the role of continuous dural suture in endoscopic surgery. A total of 79 adult patients, all of whom had grade 3 CSF leakage during surgery, were compared between patients who underwent and did not undergo endoscopic dural suture after tumor resection. 79 adult patients had Esposito Grade 3 high-flow CSF leakage during surgery. Po-CSF leakage occurred in 10 patients (12.7%). Po-CSF leakage occurred in 1 of 36 patients who underwent intraoperative dural suture, while po-CSF leakage occurred in 9 of 43 patients who did not undergo intraoperative dural suture (*p* = 0.016). Regression analysis showed that dural suture significantly reduced the incidence of po-CSF leakage (*p* = 0.049, OR 0.108, 95% CI 0.013–0.899). Fascia Lata was chosen as the repair material because it is more plastic than other dural alternatives and can reduce financial costs for patients. We also examined the learning curve for dural suturing under EETSS. When this procedure was first implemented, it took more than 90 min to complete closure with dural suturing. After gaining experience with 15–20 cases,2 veteran surgeons could finish the suturing procedure within 30 min (14). However, we do not deny the important role of the principle of multi-layer repair in skull base reconstruction, which is also the general guiding direction in our repair process. In craniotomy operations, the dura is typically closed as tightly as possible and covered with fibrin glue; any dural defects are closed with fascia or galea. In EES, the dura should be closed tightly in regions where CSF may easily leak when possible. Ishikawa et al. described three intraoperative dural continuous suture techniques for CSF leak repair during EES, which reduced the frequency of lumbar drainage (15). Kim et al. reported an intraoperative clipping technique that was reliable for repairing intraoperative CSF leakage (16). With our strategy, the original leak is sealed in any patient with visible CSF leakage (grade 1–3) using suturing or placement of a fat graft or “bathtub plug.” Our data show that this approach decreases the incidence of CSF leakage and the need for lumbar drainage.

In patients with grade 3 CSF leakage, we believe that current methods still have some limitations, and suture fascia through the nasal passage at the base of the skull is indeed difficult. However, training in proper dural suture can allow neurosurgeons to perform surgery safely and smoothly in this small space. Ishikawa et al. reported the good results achieved by the implementation of skull base suture in their center (15). As mentioned above, we believe that through the accumulation of experience, the operation time can be further shortened.

Lumbar drainage reduces CSF pulsations (17), which contribute to postoperative CSF leakage. Although effective, lumbar drainage is associated with risks of nerve injury and infection and requires prolonged bedrest, which increases risks of muscle wasting and deep venous thrombosis.

### Eliminating dead space

Our second principle of repair, elimination of dead space, aims to reduce the effect of CSF pulsations on the sellar floor. We primarily use fat tissue to achieve this in patients with visible CSF leakage (grades 1–3), which is suitable because it can adhere to the surrounding tissue. In addition, fat is hydrophobic, light in weight, and will not compress local tissue. However, it takes several days or weeks for adherence to occur; therefore, patients with a grade 3 CSF leak may need additional fixation. We suture dura for fixation, ensuring close contact between the dura and fat to prevent CSF leakage. This appears to ensure rapid and tight adhesion. Previously reported repair methods emphasize the principle of filling the sella (9, 10). For grade 0 CSF leaks, we typically use gelatin sponges to eliminate dead space; however, if the diaphragma sellae is transparent, autologous fat is used.

### Providing blood supply

Providing blood supply is our third principle of skull base reconstruction. Vascularized nasoseptal flaps were first described in 2006 (8) and have become important in skull base reconstruction. Cruttenden et al. reported that the septum nasopharyngeal flap is a safe and ideal material for skull base reconstruction after extensive intranasal slope tumor resection (18). We also use a nasoseptal mucosal flap to provide blood supply, particularly in patients with grade 3 CSF leakage. Our routine use of this flap in these patients may explain our low postoperative CSF leak rate. Although it is reliable for preventing CSF leakage, its use has been associated with olfactory dysfunction and impaired mucociliary clearance (19). We therefore try to minimize use of the nasoseptal flap. In our experience, it is not necessary unless a large dural defect is present. When we do use it, we try to avoid damaging the olfactory area when creating the flap.

### Early ambulation, rapid recovery

In our center, lumbar drainage was not routinely used in all patients after surgery, and patients were asked to rest in a semi-sitting position on the postoperative day to reduce the pressure on the skull base defect. On the first postoperative day, patients were encouraged to get out of bed. This is consistent with “Flash” in the “3F” intermediate strategy, and we believe that early ambulation is an important measure to accelerate postoperative recovery.

### Limitations of the study

Retrospective analysis is the main limitation of this study. Moreover, the manuscript was designed to clarify the philosophical issue of strategy and specific reconstruction methods for skull base reconstruction, and there was no control group, so statistical analysis was not applicable to this study. Short follow-up time was another limitation. Future prospective studies should be selected to further clarify the effectiveness of this reconstruction strategy.

## Conclusion

The principles of sealing the original leak, eliminating dead space, providing blood supply and early ambulation are key in skull base reconstruction after EES. Individualization of these principles can significantly reduce the incidence of postoperative CSF leakage and intracranial infection and reduce the use of lumbar CSF drainage. Skull base suture technique is safe and effective in patients with high-flow cerebrospinal fluid leaks.

## Data Availability

The original contributions presented in the study are included in the article/**Supplementary Material**, further inquiries can be directed to the corresponding authors.
